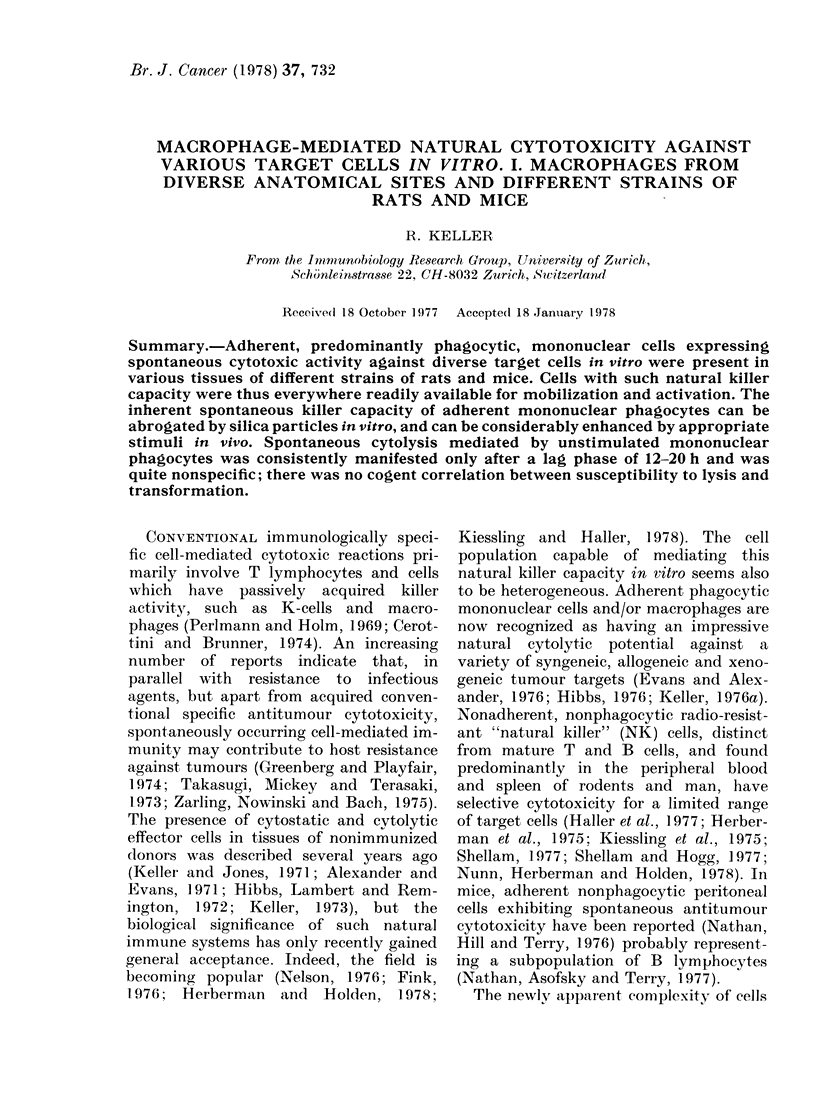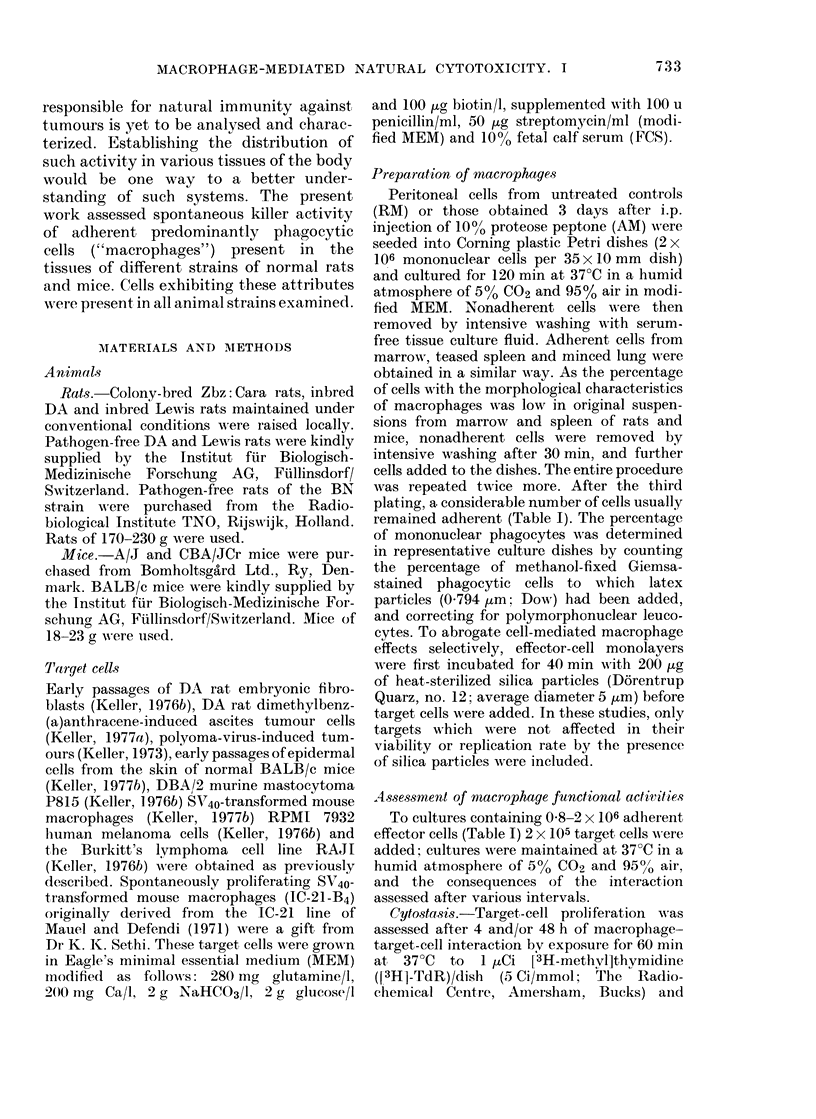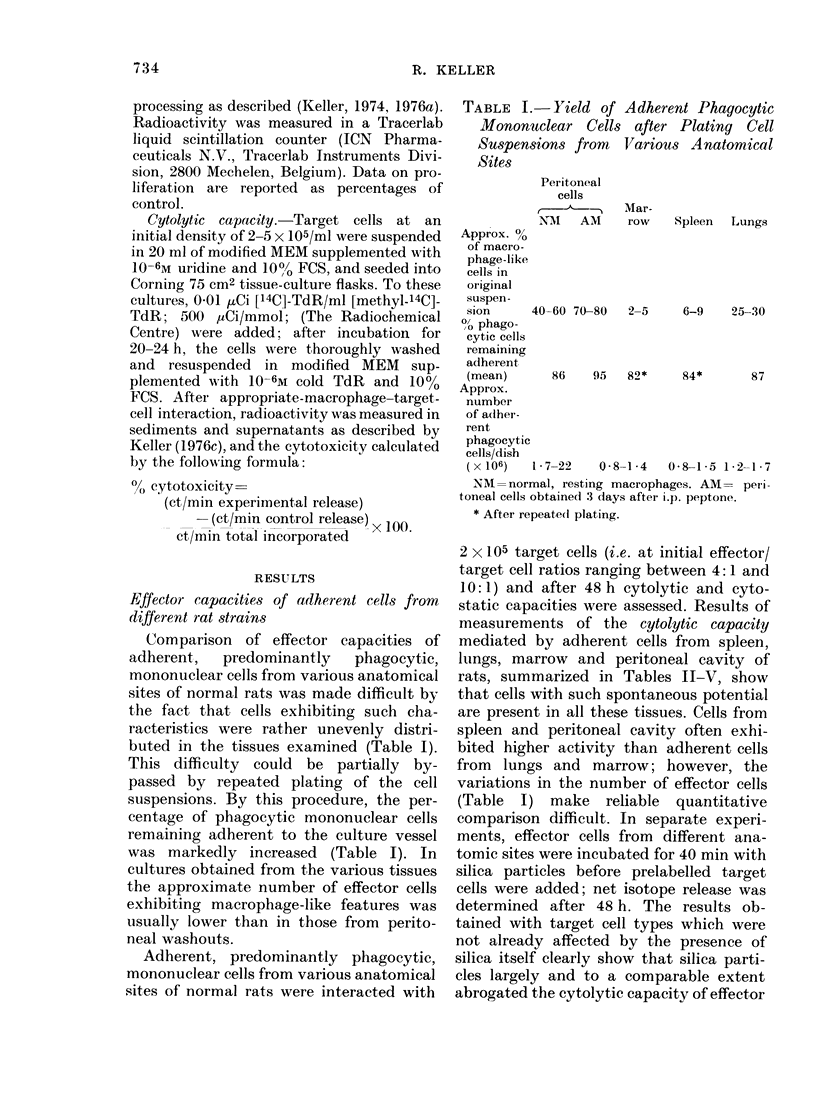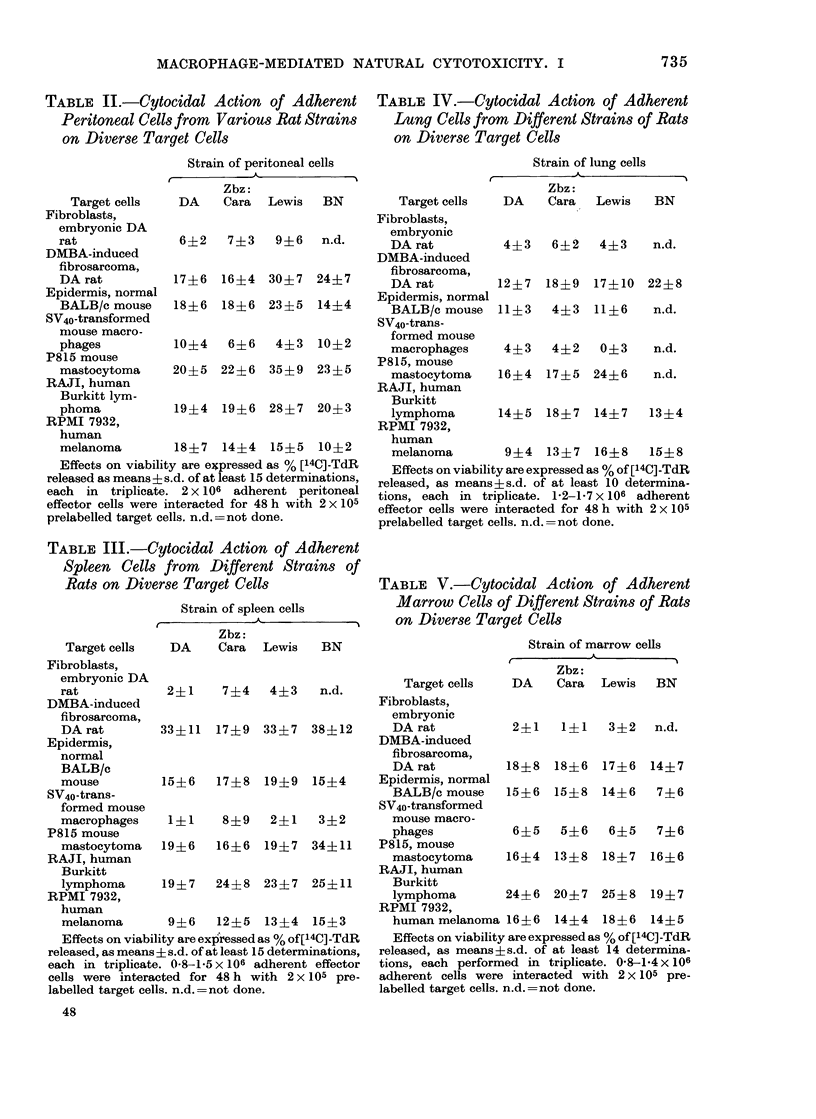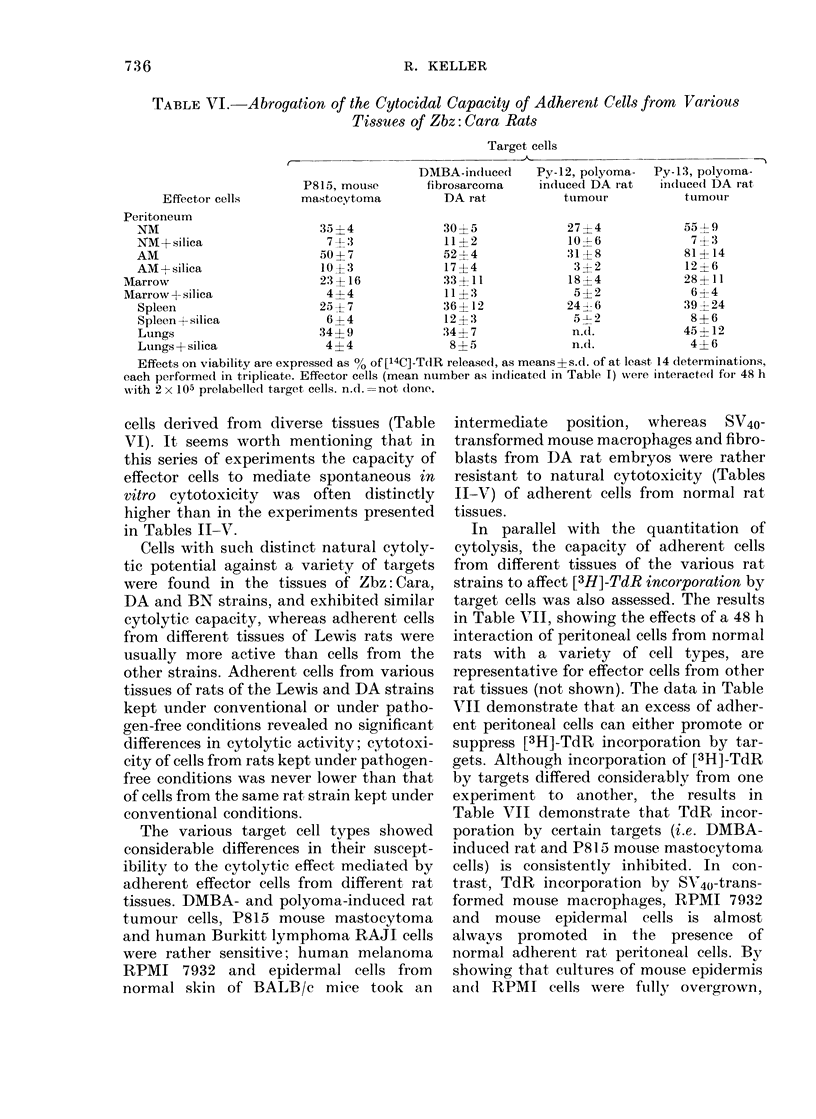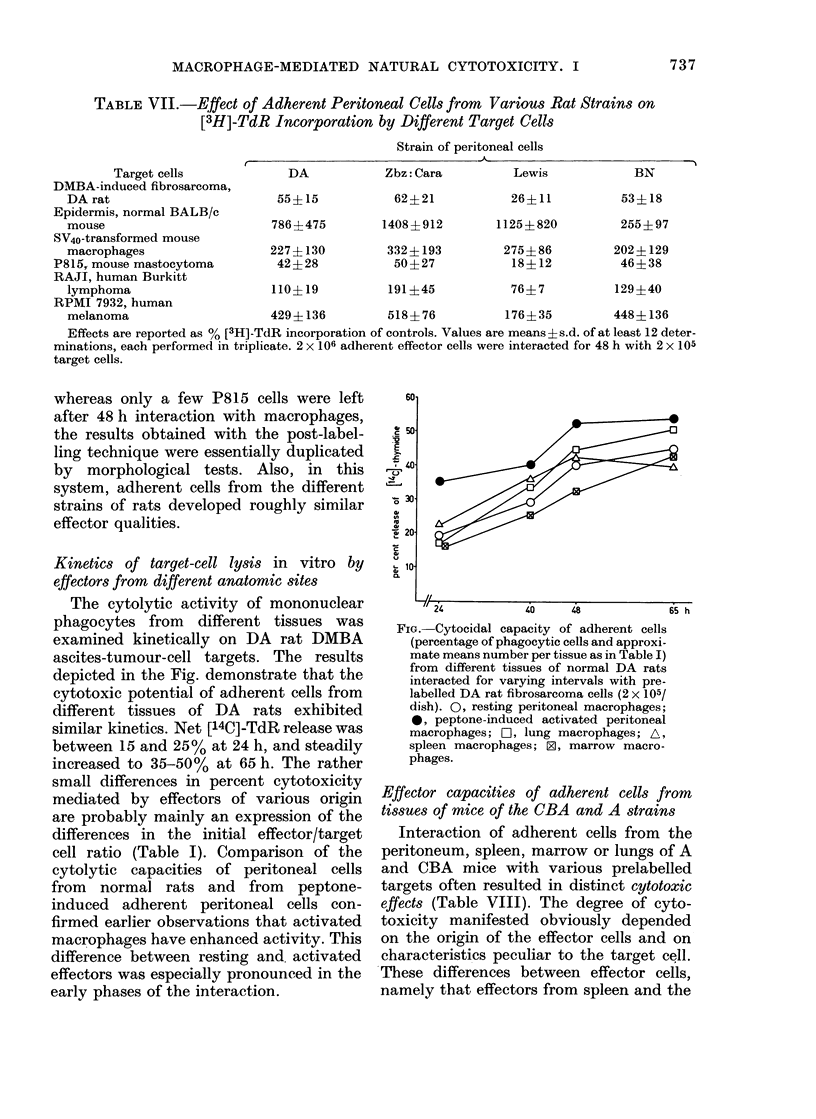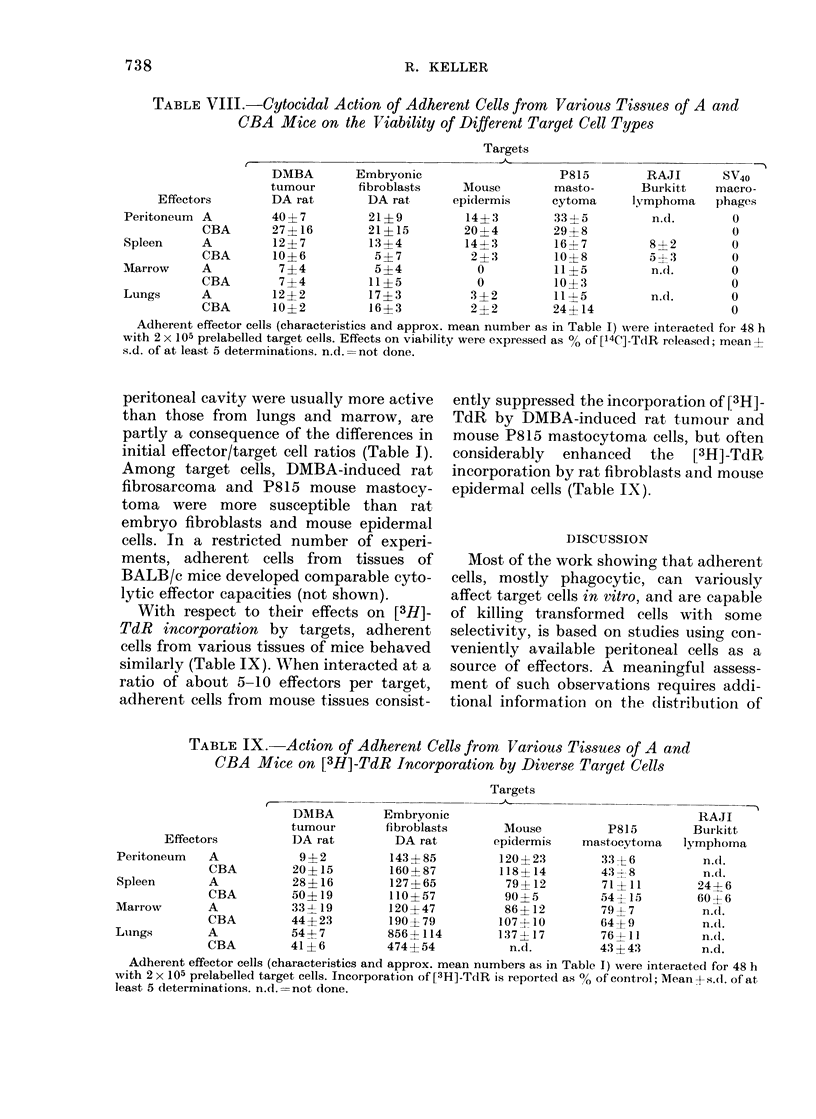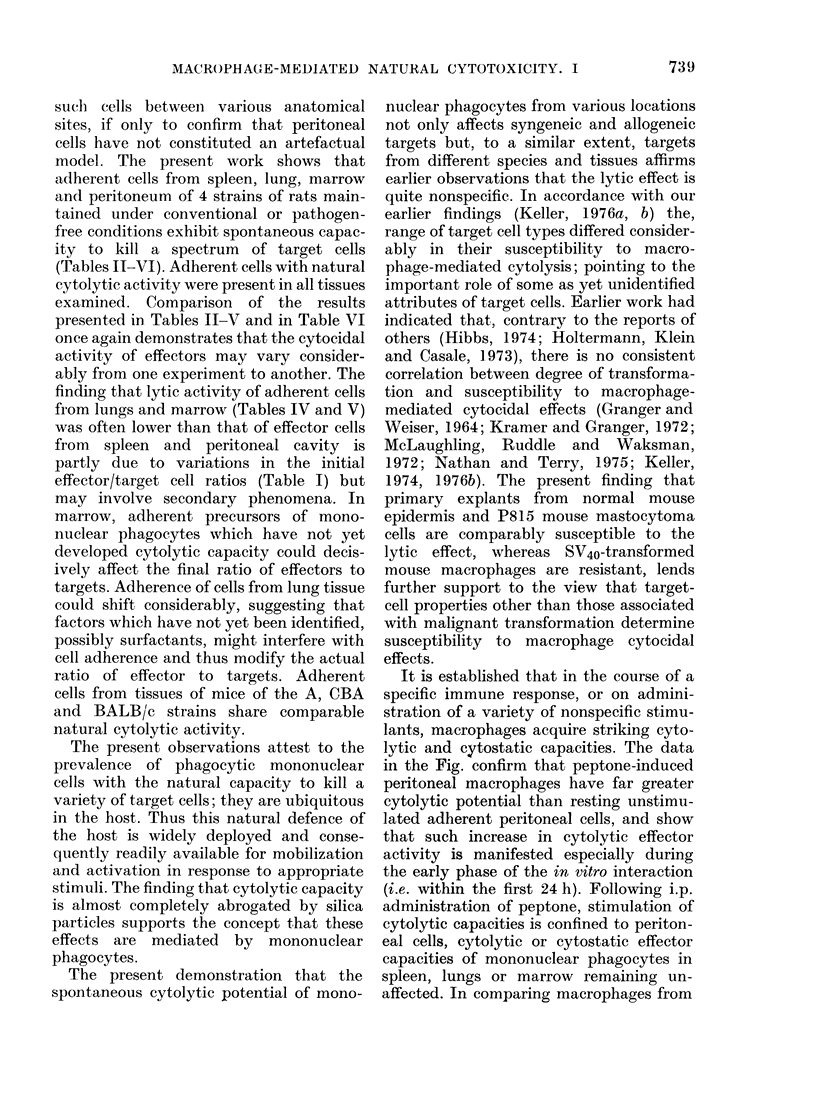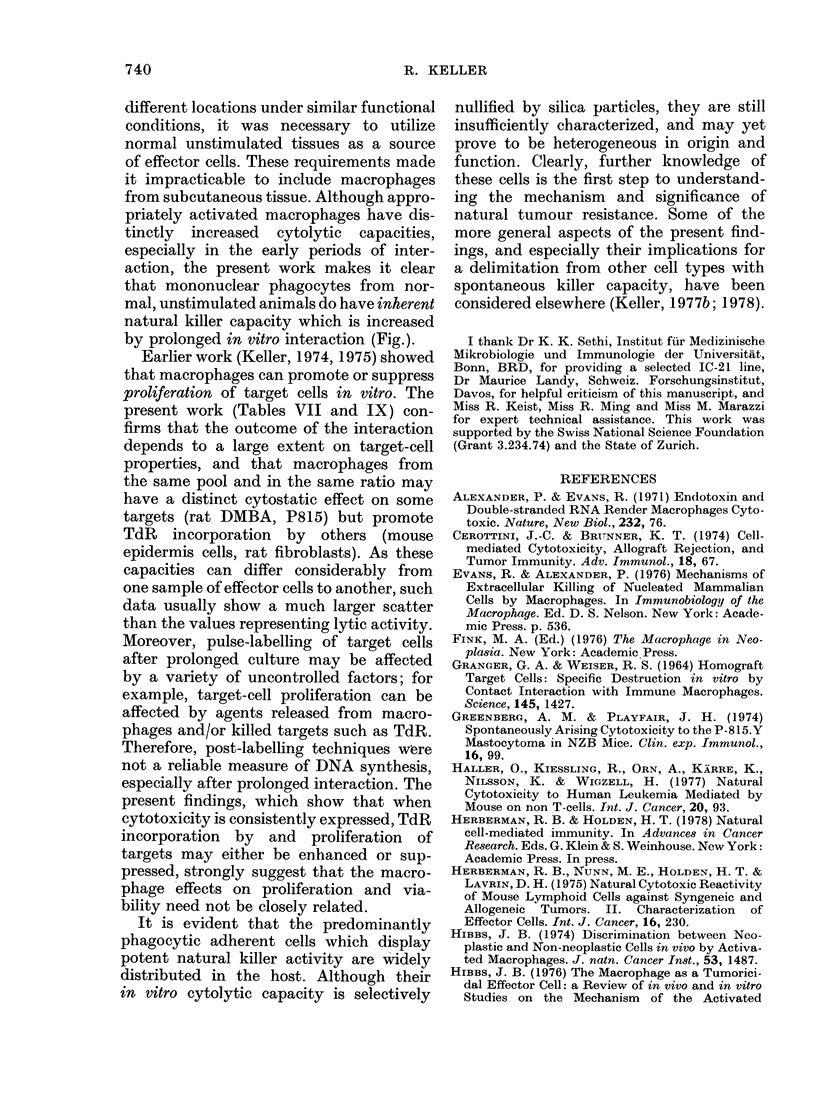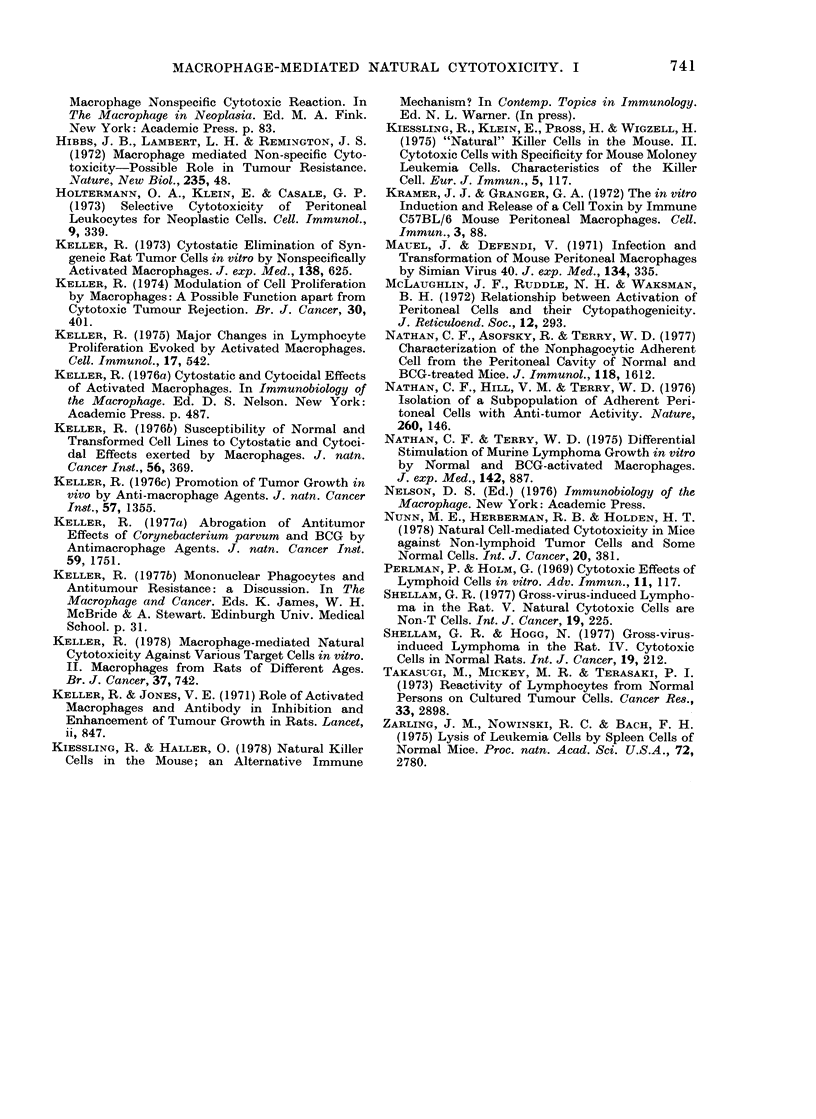# Macrophage-mediated natrual cytotoxicity against various target cells in vitro. I. Macrophages from diverse anatomical sites and different strains of rats and mice.

**DOI:** 10.1038/bjc.1978.111

**Published:** 1978-05

**Authors:** R. Keller

## Abstract

Adherent, predominantly phagocytic, mononuclear cells expressing spontaneous cytotoxic activity against diverse target cells in vitro were present in various tissues of different strains of rats and mice. Cells with such natural killer capacity were thus everywhere readily available for mobilization and activation. The inherent spontaneous killer capacity of adherent mononuclear phagocytes can be abrogated by silica particles in vitro, and can be considerably enhanced by appropriate stimuli in vivo. Spontaneous cytolysis mediated by unstimulated mononuclear phagocytes was consistently manifested only after a lag phase of 12-20 h and was quite nonspecific; there was no cogent correlation between susceptibility to lysis and transformation.


*Br. J. Cancer* (1978) **37**, 732

### MACROPHAGE-MEDIATED NATURAL CYTOTOXICITY AGAINST VARIOUS TARGET CELLS *IN VITRO*. I. MACROPHAGES FROM DIVERSE ANATOMICAL SITES AND DIFFERENT STRAINS OF RATS AND MICE

R. KELLER

*From the Immunobiology Research Group, University of Zurich, Schönleinstrasse 22, CH-8032 Zurich, Switzerland*

Received 18 October 1977 Accepted 18 January 1978

**Summary.—Adherent, predominantly phagocytic, mononuclear cells expressing spontaneous cytotoxic activity against diverse target cells *in vitro* were present in various tissues of different strains of rats and mice. Cells with such natural killer capacity were thus everywhere readily available for mobilization and activation. The inherent spontaneous killer capacity of adherent mononuclear phagocytes can be abrogated by silica particles *in vitro*, and can be considerably enhanced by appropriate stimuli *in vivo*. Spontaneous cytolysis mediated by unstimulated mononuclear phagocytes was consistently manifested only after a lag phase of 12–20 h and was quite nonspecific; there was no cogent correlation between susceptibility to lysis and transformation.**

CONVENTIONAL immunologically specific cell-mediated cytotoxic reactions primarily involve T lymphocytes and cells which have passively acquired killer activity, such as K-cells and macrophages (Perlmann and Holm, 1969; Cerottini and Brunner, 1974). An increasing number of reports indicate that, in parallel with resistance to infectious agents, but apart from acquired conventional specific antitumour cytotoxicity, spontaneously occurring cell-mediated immunity may contribute to host resistance against tumours (Greenberg and Playfair, 1974; Takasugi, Mickey and Terasaki, 1973; Zarling, Nowinski and Bach, 1975). The presence of cytostatic and cytolytic effector cells in tissues of nonimmunized donors was described several years ago (Keller and Jones, 1971; Alexander and Evans, 1971; Hibbs, Lambert and Remington, 1972; Keller, 1973), but the biological significance of such natural immune systems has only recently gained general acceptance. Indeed, the field is becoming popular (Nelson, 1976; Fink, 1976; Herberman and Holden, 1978; Kiessling and Haller, 1978). The cell population capable of mediating this natural killer capacity *in vitro* seems also to be heterogeneous. Adherent phagocytic mononuclear cells and/or macrophages are now recognized as having an impressive natural cytolytic potential against a variety of syngeneic, allogeneic and xenogeneic tumour targets (Evans and Alexander, 1976; Hibbs, 1976; Keller, 1976*a*). Nonadherent, nonphagocytic radio-resistant "natural killer" (NK) cells, distinct from mature T and B cells, and found predominantly in the peripheral blood and spleen of rodents and man, have selective cytotoxicity for a limited range of target cells (Haller *et al.*, 1977; Herberman *et al.*, 1975; Kiessling *et al.*, 1975; Shellam, 1977; Shellam and Hogg, 1977; Nunn, Herberman and Holden, 1978). In mice, adherent nonphagocytic peritoneal cells exhibiting spontaneous antitumour cytotoxicity have been reported (Nathan, Hill and Terry, 1976) probably representing a subpopulation of B lymphocytes (Nathan, Asofsky and Terry, 1977).

The newly apparent complexity of cells responsible for natural immunity against tumours is yet to be analysed and characterized. Establishing the distribution of such activity in various tissues of the body would be one way to a better understanding of such systems. The present work assessed spontaneous killer activity of adherent predominantly phagocytic cells ("macrophages") present in the tissues of different strains of normal rats and mice. Cells exhibiting these attributes were present in all animal strains examined.

### MATERIALS AND METHODS

### Animals

*Rats.*—Colony-bred Zbz: Cara rats, inbred DA and inbred Lewis rats maintained under conventional conditions were raised locally. Pathogen-free DA and Lewis rats were kindly supplied by the Institut für Biologisch-Medizinische Forschung AG, Füllinsdorf/Switzerland. Pathogen-free rats of the BN strain were purchased from the Radiobiological Institute TNO, Rijswijk, Holland. Rats of 170–230 g were used.

*Mice.*—A/J and CBA/JCr mice were purchased from Bomholtsgård Ltd., Ry, Denmark. BALB/c mice were kindly supplied by the Institut für Biologisch-Medizinische Forschung AG, Füllinsdorf/Switzerland. Mice of 18–23 g were used.

### Target cells

Early passages of DA rat embryonic fibroblasts (Keller, 1976*b*), DA rat dimethylbenz-(a)anthracene-induced ascites tumour cells (Keller, 1977*a*), polyoma-virus-induced tumours (Keller, 1973), early passages of epidermal cells from the skin of normal BALB/c mice (Keller, 1977*b*), DBA/2 murine mastocytoma P815 (Keller, 1976*b*) $SV_{40}$-transformed mouse macrophages (Keller, 1977*b*) RPMI 7932 human melanoma cells (Keller, 1976*b*) and the Burkitt's lymphoma cell line RAJI (Keller, 1976*b*) were obtained as previously described. Spontaneously proliferating $SV_{40}$-transformed mouse macrophages (IC-21-$B_4$) originally derived from the IC-21 line of Mauel and Defendi (1971) were a gift from Dr K. K. Sethi. These target cells were grown in Eagle's minimal essential medium (MEM) modified as follows: 280 mg glutamine/l, 200 mg Ca/l, 2 g $NaHCO_3$/l, 2 g glucose/l and 100 $\mu$g biotin/l, supplemented with 100 u penicillin/ml, 50 $\mu$g streptomycin/ml (modified MEM) and 10% fetal calf serum (FCS).

### Preparation of macrophages

Peritoneal cells from untreated controls (RM) or those obtained 3 days after i.p. injection of 10% proteose peptone (AM) were seeded into Corning plastic Petri dishes ($2 \times 10^6$ mononuclear cells per $35 \times 10$ mm dish) and cultured for 120 min at 37°C in a humid atmosphere of 5% $CO_2$ and 95% air in modified MEM. Nonadherent cells were then removed by intensive washing with serum-free tissue culture fluid. Adherent cells from marrow, teased spleen and minced lung were obtained in a similar way. As the percentage of cells with the morphological characteristics of macrophages was low in original suspensions from marrow and spleen of rats and mice, nonadherent cells were removed by intensive washing after 30 min, and further cells added to the dishes. The entire procedure was repeated twice more. After the third plating, a considerable number of cells usually remained adherent (Table I). The percentage of mononuclear phagocytes was determined in representative culture dishes by counting the percentage of methanol-fixed Giemsa-stained phagocytic cells to which latex particles (0·794 $\mu$m; Dow) had been added, and correcting for polymorphonuclear leucocytes. To abrogate cell-mediated macrophage effects selectively, effector-cell monolayers were first incubated for 40 min with 200 $\mu$g of heat-sterilized silica particles (Dörentrup Quarz, no. 12; average diameter 5 $\mu$m) before target cells were added. In these studies, only targets which were not affected in their viability or replication rate by the presence of silica particles were included.

### Assessment of macrophage functional activities

To cultures containing $0·8–2 \times 10^6$ adherent effector cells (Table I) $2 \times 10^5$ target cells were added; cultures were maintained at 37°C in a humid atmosphere of 5% $CO_2$ and 95% air, and the consequences of the interaction assessed after various intervals.

*Cytostasis.*—Target-cell proliferation was assessed after 4 and/or 48 h of macrophage–target-cell interaction by exposure for 60 min at 37°C to 1 $\mu$Ci [$^3$H-methyl]thymidine ([$^3$H]-TdR)/dish (5 Ci/mmol; The Radiochemical Centre, Amersham, Bucks) and processing as described (Keller, 1974, 1976*a*). Radioactivity was measured in a Tracerlab liquid scintillation counter (ICN Pharmaceuticals N.V., Tracerlab Instruments Division, 2800 Mechelen, Belgium). Data on proliferation are reported as percentages of control.

*Cytolytic capacity.*—Target cells at an initial density of $2\text{–}5 \times 10^5$/ml were suspended in 20 ml of modified MEM supplemented with $10^{-6}$M uridine and 10% FCS, and seeded into Corning 75 cm² tissue-culture flasks. To these cultures, 0·01 μCi [$^{14}$C]-TdR/ml [methyl-$^{14}$C]-TdR; 500 μCi/mmol; (The Radiochemical Centre) were added; after incubation for 20–24 h, the cells were thoroughly washed and resuspended in modified MEM supplemented with $10^{-6}$M cold TdR and 10% FCS. After appropriate-macrophage–target-cell interaction, radioactivity was measured in sediments and supernatants as described by Keller (1976*c*), and the cytotoxicity calculated by the following formula:

$$\%\ \text{cytotoxicity} = \frac{(\text{ct/min experimental release}) - (\text{ct/min control release})}{\text{ct/min total incorporated}} \times 100.$$

RESULTS

*Effector capacities of adherent cells from different rat strains*

Comparison of effector capacities of adherent, predominantly phagocytic, mononuclear cells from various anatomical sites of normal rats was made difficult by the fact that cells exhibiting such characteristics were rather unevenly distributed in the tissues examined (Table I). This difficulty could be partially bypassed by repeated plating of the cell suspensions. By this procedure, the percentage of phagocytic mononuclear cells remaining adherent to the culture vessel was markedly increased (Table I). In cultures obtained from the various tissues the approximate number of effector cells exhibiting macrophage-like features was usually lower than in those from peritoneal washouts.

Adherent, predominantly phagocytic, mononuclear cells from various anatomical sites of normal rats were interacted with

TABLE I.—*Yield of Adherent Phagocytic Mononuclear Cells after Plating Cell Suspensions from Various Anatomical Sites*

| | Peritoneal cells NM | Peritoneal cells AM | Marrow | Spleen | Lungs |
|---|---|---|---|---|---|
| Approx. % of macrophage-like cells in original suspension | 40–60 | 70–80 | 2–5 | 6–9 | 25–30 |
| % phagocytic cells remaining adherent (mean) | 86 | 95 | 82* | 84* | 87 |
| Approx. number of adherent phagocytic cells/dish (×10⁶) | 1·7–22 | | 0·8–1·4 | 0·8–1·5 | 1·2–1·7 |

NM = normal, resting macrophages. AM = peritoneal cells obtained 3 days after i.p. peptone.

* After repeated plating.

$2 \times 10^5$ target cells (*i.e.* at initial effector/target cell ratios ranging between 4:1 and 10:1) and after 48 h cytolytic and cytostatic capacities were assessed. Results of measurements of the *cytolytic capacity* mediated by adherent cells from spleen, lungs, marrow and peritoneal cavity of rats, summarized in Tables II–V, show that cells with such spontaneous potential are present in all these tissues. Cells from spleen and peritoneal cavity often exhibited higher activity than adherent cells from lungs and marrow; however, the variations in the number of effector cells (Table I) make reliable quantitative comparison difficult. In separate experiments, effector cells from different anatomic sites were incubated for 40 min with silica particles before prelabelled target cells were added; net isotope release was determined after 48 h. The results obtained with target cell types which were not already affected by the presence of silica itself clearly show that silica particles largely and to a comparable extent abrogated the cytolytic capacity of effector

TABLE II.—*Cytocidal Action of Adherent Peritoneal Cells from Various Rat Strains on Diverse Target Cells*

| | Strain of peritoneal cells | | | |
|---|---|---|---|---|
| Target cells | DA | Zbz: Cara | Lewis | BN |
| Fibroblasts, embryonic DA rat | 6±2 | 7±3 | 9±6 | n.d. |
| DMBA-induced fibrosarcoma, DA rat | 17±6 | 16±4 | 30±7 | 24±7 |
| Epidermis, normal BALB/c mouse | 18±6 | 18±6 | 23±5 | 14±4 |
| $SV_{40}$-transformed mouse macrophages | 10±4 | 6±6 | 4±3 | 10±2 |
| P815 mouse mastocytoma | 20±5 | 22±6 | 35±9 | 23±5 |
| RAJI, human Burkitt lymphoma | 19±4 | 19±6 | 28±7 | 20±3 |
| RPMI 7932, human melanoma | 18±7 | 14±4 | 15±5 | 10±2 |

Effects on viability are expressed as % [$^{14}$C]-TdR released as means±s.d. of at least 15 determinations, each in triplicate. $2\times10^6$ adherent peritoneal effector cells were interacted for 48 h with $2\times10^5$ prelabelled target cells. n.d.=not done.

TABLE III.—*Cytocidal Action of Adherent Spleen Cells from Different Strains of Rats on Diverse Target Cells*

| | Strain of spleen cells | | | |
|---|---|---|---|---|
| Target cells | DA | Zbz: Cara | Lewis | BN |
| Fibroblasts, embryonic DA rat | 2±1 | 7±4 | 4±3 | n.d. |
| DMBA-induced fibrosarcoma, DA rat | 33±11 | 17±9 | 33±7 | 38±12 |
| Epidermis, normal BALB/c mouse | 15±6 | 17±8 | 19±9 | 15±4 |
| $SV_{40}$-transformed mouse macrophages | 1±1 | 8±9 | 2±1 | 3±2 |
| P815 mouse mastocytoma | 19±6 | 16±6 | 19±7 | 34±11 |
| RAJI, human Burkitt lymphoma | 19±7 | 24±8 | 23±7 | 25±11 |
| RPMI 7932, human melanoma | 9±6 | 12±5 | 13±4 | 15±3 |

Effects on viability are expressed as % of[$^{14}$C]-TdR released, as means±s.d. of at least 15 determinations, each in triplicate. 0·8–1·5$\times10^6$ adherent effector cells were interacted for 48 h with $2\times10^5$ prelabelled target cells. n.d.=not done.

TABLE IV.—*Cytocidal Action of Adherent Lung Cells from Different Strains of Rats on Diverse Target Cells*

| | Strain of lung cells | | | |
|---|---|---|---|---|
| Target cells | DA | Zbz: Cara | Lewis | BN |
| Fibroblasts, embryonic DA rat | 4±3 | 6±2 | 4±3 | n.d. |
| DMBA-induced fibrosarcoma, DA rat | 12±7 | 18±9 | 17±10 | 22±8 |
| Epidermis, normal BALB/c mouse | 11±3 | 4±3 | 11±6 | n.d. |
| $SV_{40}$-transformed mouse macrophages | 4±3 | 4±2 | 0±3 | n.d. |
| P815, mouse mastocytoma | 16±4 | 17±5 | 24±6 | n.d. |
| RAJI, human Burkitt lymphoma | 14±5 | 18±7 | 14±7 | 13±4 |
| RPMI 7932, human melanoma | 9±4 | 13±7 | 16±8 | 15±8 |

Effects on viability are expressed as % of [$^{14}$C]-TdR released, as means±s.d. of at least 10 determinations, each in triplicate. 1·2–1·7$\times10^6$ adherent effector cells were interacted for 48 h with $2\times10^5$ prelabelled target cells. n.d.=not done.

TABLE V.—*Cytocidal Action of Adherent Marrow Cells of Different Strains of Rats on Diverse Target Cells*

| | Strain of marrow cells | | | |
|---|---|---|---|---|
| Target cells | DA | Zbz: Cara | Lewis | BN |
| Fibroblasts, embryonic DA rat | 2±1 | 1±1 | 3±2 | n.d. |
| DMBA-induced fibrosarcoma, DA rat | 18±8 | 18±6 | 17±6 | 14±7 |
| Epidermis, normal BALB/c mouse | 15±6 | 15±8 | 14±6 | 7±6 |
| $SV_{40}$-transformed mouse macrophages | 6±5 | 5±6 | 6±5 | 7±6 |
| P815, mouse mastocytoma | 16±4 | 13±8 | 18±7 | 16±6 |
| RAJI, human Burkitt lymphoma | 24±6 | 20±7 | 25±8 | 19±7 |
| RPMI 7932, human melanoma | 16±6 | 14±4 | 18±6 | 14±5 |

Effects on viability are expressed as % of[$^{14}$C]-TdR released, as means±s.d. of at least 14 determinations, each performed in triplicate. 0·8–1·4$\times10^6$ adherent cells were interacted with $2\times10^5$ prelabelled target cells. n.d.=not done.

TABLE VI.—*Abrogation of the Cytocidal Capacity of Adherent Cells from Various Tissues of Zbz: Cara Rats*

| Effector cells | Target cells: P815, mouse mastocytoma | DMBA-induced fibrosarcoma DA rat | Py-12, polyoma-induced DA rat tumour | Py-13, polyoma-induced DA rat tumour |
|---|---|---|---|---|
| Peritoneum | | | | |
| NM | 35±4 | 30±5 | 27±4 | 55±9 |
| NM+silica | 7±3 | 11±2 | 10±6 | 7±3 |
| AM | 50±7 | 52±4 | 31±8 | 81±14 |
| AM+silica | 10±3 | 17±4 | 3±2 | 12±6 |
| Marrow | 23±16 | 33±11 | 18±4 | 28±11 |
| Marrow+silica | 4±4 | 11±3 | 5±2 | 6±4 |
| Spleen | 25±7 | 36±12 | 24±6 | 39±24 |
| Spleen+silica | 6±4 | 12±3 | 5±2 | 8±6 |
| Lungs | 34±9 | 34±7 | n.d. | 45±12 |
| Lungs+silica | 4±4 | 8±5 | n.d. | 4±6 |

Effects on viability are expressed as % of [$^{14}$C]-TdR released, as means±s.d. of at least 14 determinations, each performed in triplicate. Effector cells (mean number as indicated in Table I) were interacted for 48 h with $2\times10^5$ prelabelled target cells. n.d.=not done.

cells derived from diverse tissues (Table VI). It seems worth mentioning that in this series of experiments the capacity of effector cells to mediate spontaneous *in vitro* cytotoxicity was often distinctly higher than in the experiments presented in Tables II–V.

Cells with such distinct natural cytolytic potential against a variety of targets were found in the tissues of Zbz: Cara, DA and BN strains, and exhibited similar cytolytic capacity, whereas adherent cells from different tissues of Lewis rats were usually more active than cells from the other strains. Adherent cells from various tissues of rats of the Lewis and DA strains kept under conventional or under pathogen-free conditions revealed no significant differences in cytolytic activity; cytotoxicity of cells from rats kept under pathogen-free conditions was never lower than that of cells from the same rat strain kept under conventional conditions.

The various target cell types showed considerable differences in their susceptibility to the cytolytic effect mediated by adherent effector cells from different rat tissues. DMBA- and polyoma-induced rat tumour cells, P815 mouse mastocytoma and human Burkitt lymphoma RAJI cells were rather sensitive; human melanoma RPMI 7932 and epidermal cells from normal skin of BALB/c mice took an intermediate position, whereas $SV_{40}$-transformed mouse macrophages and fibroblasts from DA rat embryos were rather resistant to natural cytotoxicity (Tables II–V) of adherent cells from normal rat tissues.

In parallel with the quantitation of cytolysis, the capacity of adherent cells from different tissues of the various rat strains to affect [$^3$*H*]-*TdR incorporation* by target cells was also assessed. The results in Table VII, showing the effects of a 48 h interaction of peritoneal cells from normal rats with a variety of cell types, are representative for effector cells from other rat tissues (not shown). The data in Table VII demonstrate that an excess of adherent peritoneal cells can either promote or suppress [$^3$H]-TdR incorporation by targets. Although incorporation of [$^3$H]-TdR by targets differed considerably from one experiment to another, the results in Table VII demonstrate that TdR incorporation by certain targets (*i.e.* DMBA-induced rat and P815 mouse mastocytoma cells) is consistently inhibited. In contrast, TdR incorporation by $SV_{40}$-transformed mouse macrophages, RPMI 7932 and mouse epidermal cells is almost always promoted in the presence of normal adherent rat peritoneal cells. By showing that cultures of mouse epidermis and RPMI cells were fully overgrown,

TABLE VII.—*Effect of Adherent Peritoneal Cells from Various Rat Strains on [³H]-TdR Incorporation by Different Target Cells*

| | Strain of peritoneal cells | | | |
|---|---|---|---|---|
| Target cells | DA | Zbz:Cara | Lewis | BN |
| DMBA-induced fibrosarcoma, DA rat | 55±15 | 62±21 | 26±11 | 53±18 |
| Epidermis, normal BALB/c mouse | 786±475 | 1408±912 | 1125±820 | 255±97 |
| $SV_{40}$-transformed mouse macrophages | 227±130 | 332±193 | 275±86 | 202±129 |
| P815, mouse mastocytoma | 42±28 | 50±27 | 18±12 | 46±38 |
| RAJI, human Burkitt lymphoma | 110±19 | 191±45 | 76±7 | 129±40 |
| RPMI 7932, human melanoma | 429±136 | 518±76 | 176±35 | 448±136 |

Effects are reported as % [$^3$H]-TdR incorporation of controls. Values are means±s.d. of at least 12 determinations, each performed in triplicate. $2 \times 10^6$ adherent effector cells were interacted for 48 h with $2 \times 10^5$ target cells.

whereas only a few P815 cells were left after 48 h interaction with macrophages, the results obtained with the post-labelling technique were essentially duplicated by morphological tests. Also, in this system, adherent cells from the different strains of rats developed roughly similar effector qualities.

*Kinetics of target-cell lysis* in vitro *by effectors from different anatomic sites*

The cytolytic activity of mononuclear phagocytes from different tissues was examined kinetically on DA rat DMBA ascites-tumour-cell targets. The results depicted in the Fig. demonstrate that the cytotoxic potential of adherent cells from different tissues of DA rats exhibited similar kinetics. Net [$^{14}$C]-TdR release was between 15 and 25% at 24 h, and steadily increased to 35–50% at 65 h. The rather small differences in percent cytotoxicity mediated by effectors of various origin are probably mainly an expression of the differences in the initial effector/target cell ratio (Table I). Comparison of the cytolytic capacities of peritoneal cells from normal rats and from peptone-induced adherent peritoneal cells confirmed earlier observations that activated macrophages have enhanced activity. This difference between resting and activated effectors was especially pronounced in the early phases of the interaction.

FIG.—Cytocidal capacity of adherent cells (percentage of phagocytic cells and approximate means number per tissue as in Table I) from different tissues of normal DA rats interacted for varying intervals with prelabelled DA rat fibrosarcoma cells ($2 \times 10^5$/dish). ○, resting peritoneal macrophages; ●, peptone-induced activated peritoneal macrophages; □, lung macrophages; △, spleen macrophages; ⊠, marrow macrophages.

*Effector capacities of adherent cells from tissues of mice of the CBA and A strains*

Interaction of adherent cells from the peritoneum, spleen, marrow or lungs of A and CBA mice with various prelabelled targets often resulted in distinct *cytotoxic effects* (Table VIII). The degree of cytotoxicity manifested obviously depended on the origin of the effector cells and on characteristics peculiar to the target cell. These differences between effector cells, namely that effectors from spleen and the

TABLE VIII.—*Cytocidal Action of Adherent Cells from Various Tissues of A and CBA Mice on the Viability of Different Target Cell Types*

| Effectors | | DMBA tumour DA rat | Embryonic fibroblasts DA rat | Mouse epidermis | P815 masto-cytoma | RAJI Burkitt lymphoma | $SV_{40}$ macro-phages |
|---|---|---|---|---|---|---|---|
| Peritoneum | A | 40±7 | 21±9 | 14±3 | 33±5 | n.d. | 0 |
| | CBA | 27±16 | 21±15 | 20±4 | 29±8 | | 0 |
| Spleen | A | 12±7 | 13±4 | 14±3 | 16±7 | 8±2 | 0 |
| | CBA | 10±6 | 5±7 | 2±3 | 10±8 | 5±3 | 0 |
| Marrow | A | 7±4 | 5±4 | 0 | 11±5 | n.d. | 0 |
| | CBA | 7±4 | 11±5 | 0 | 10±3 | | 0 |
| Lungs | A | 12±2 | 17±3 | 3±2 | 11±5 | n.d. | 0 |
| | CBA | 10±2 | 16±3 | 2±2 | 24±14 | | 0 |

Adherent effector cells (characteristics and approx. mean number as in Table I) were interacted for 48 h with $2 \times 10^5$ prelabelled target cells. Effects on viability were expressed as % of [$^{14}$C]-TdR released; mean ± s.d. of at least 5 determinations. n.d. = not done.

peritoneal cavity were usually more active than those from lungs and marrow, are partly a consequence of the differences in initial effector/target cell ratios (Table I). Among target cells, DMBA-induced rat fibrosarcoma and P815 mouse mastocytoma were more susceptible than rat embryo fibroblasts and mouse epidermal cells. In a restricted number of experiments, adherent cells from tissues of BALB/c mice developed comparable cytolytic effector capacities (not shown).

With respect to their effects on [$^3H$]-*TdR incorporation* by targets, adherent cells from various tissues of mice behaved similarly (Table IX). When interacted at a ratio of about 5–10 effectors per target, adherent cells from mouse tissues consistently suppressed the incorporation of [$^3$H]-TdR by DMBA-induced rat tumour and mouse P815 mastocytoma cells, but often considerably enhanced the [$^3$H]-TdR incorporation by rat fibroblasts and mouse epidermal cells (Table IX).

### DISCUSSION

Most of the work showing that adherent cells, mostly phagocytic, can variously affect target cells *in vitro*, and are capable of killing transformed cells with some selectivity, is based on studies using conveniently available peritoneal cells as a source of effectors. A meaningful assessment of such observations requires additional information on the distribution of

TABLE IX.—*Action of Adherent Cells from Various Tissues of A and CBA Mice on [$^3H$]-TdR Incorporation by Diverse Target Cells*

| Effectors | | DMBA tumour DA rat | Embryonic fibroblasts DA rat | Mouse epidermis | P815 mastocytoma | RAJI Burkitt lymphoma |
|---|---|---|---|---|---|---|
| Peritoneum | A | 9±2 | 143±85 | 120±23 | 33±6 | n.d. |
| | CBA | 20±15 | 160±87 | 118±14 | 43±8 | n.d. |
| Spleen | A | 28±16 | 127±65 | 79±12 | 71±11 | 24±6 |
| | CBA | 50±19 | 110±57 | 90±5 | 54±15 | 60±6 |
| Marrow | A | 33±19 | 120±47 | 86±12 | 79±7 | n.d. |
| | CBA | 44±23 | 190±79 | 107±10 | 64±9 | n.d. |
| Lungs | A | 54±7 | 856±114 | 137±17 | 76±11 | n.d. |
| | CBA | 41±6 | 474±54 | n.d. | 43±43 | n.d. |

Adherent effector cells (characteristics and approx. mean numbers as in Table I) were interacted for 48 h with $2 \times 10^5$ prelabelled target cells. Incorporation of [$^3$H]-TdR is reported as % of control; Mean ± s.d. of at least 5 determinations. n.d. = not done.

such cells between various anatomical sites, if only to confirm that peritoneal cells have not constituted an artefactual model. The present work shows that adherent cells from spleen, lung, marrow and peritoneum of 4 strains of rats maintained under conventional or pathogen-free conditions exhibit spontaneous capacity to kill a spectrum of target cells (Tables II–VI). Adherent cells with natural cytolytic activity were present in all tissues examined. Comparison of the results presented in Tables II–V and in Table VI once again demonstrates that the cytocidal activity of effectors may vary considerably from one experiment to another. The finding that lytic activity of adherent cells from lungs and marrow (Tables IV and V) was often lower than that of effector cells from spleen and peritoneal cavity is partly due to variations in the initial effector/target cell ratios (Table I) but may involve secondary phenomena. In marrow, adherent precursors of mononuclear phagocytes which have not yet developed cytolytic capacity could decisively affect the final ratio of effectors to targets. Adherence of cells from lung tissue could shift considerably, suggesting that factors which have not yet been identified, possibly surfactants, might interfere with cell adherence and thus modify the actual ratio of effector to targets. Adherent cells from tissues of mice of the A, CBA and BALB/c strains share comparable natural cytolytic activity.

The present observations attest to the prevalence of phagocytic mononuclear cells with the natural capacity to kill a variety of target cells; they are ubiquitous in the host. Thus this natural defence of the host is widely deployed and consequently readily available for mobilization and activation in response to appropriate stimuli. The finding that cytolytic capacity is almost completely abrogated by silica particles supports the concept that these effects are mediated by mononuclear phagocytes.

The present demonstration that the spontaneous cytolytic potential of mononuclear phagocytes from various locations not only affects syngeneic and allogeneic targets but, to a similar extent, targets from different species and tissues affirms earlier observations that the lytic effect is quite nonspecific. In accordance with our earlier findings (Keller, 1976*a*, *b*) the, range of target cell types differed considerably in their susceptibility to macrophage-mediated cytolysis; pointing to the important role of some as yet unidentified attributes of target cells. Earlier work had indicated that, contrary to the reports of others (Hibbs, 1974; Holtermann, Klein and Casale, 1973), there is no consistent correlation between degree of transformation and susceptibility to macrophage-mediated cytocidal effects (Granger and Weiser, 1964; Kramer and Granger, 1972; McLaughling, Ruddle and Waksman, 1972; Nathan and Terry, 1975; Keller, 1974, 1976*b*). The present finding that primary explants from normal mouse epidermis and P815 mouse mastocytoma cells are comparably susceptible to the lytic effect, whereas $SV_{40}$-transformed mouse macrophages are resistant, lends further support to the view that target-cell properties other than those associated with malignant transformation determine susceptibility to macrophage cytocidal effects.

It is established that in the course of a specific immune response, or on administration of a variety of nonspecific stimulants, macrophages acquire striking cytolytic and cytostatic capacities. The data in the Fig. confirm that peptone-induced peritoneal macrophages have far greater cytolytic potential than resting unstimulated adherent peritoneal cells, and show that such increase in cytolytic effector activity is manifested especially during the early phase of the *in vitro* interaction (*i.e.* within the first 24 h). Following i.p. administration of peptone, stimulation of cytolytic capacities is confined to peritoneal cells, cytolytic or cytostatic effector capacities of mononuclear phagocytes in spleen, lungs or marrow remaining unaffected. In comparing macrophages from different locations under similar functional conditions, it was necessary to utilize normal unstimulated tissues as a source of effector cells. These requirements made it impracticable to include macrophages from subcutaneous tissue. Although appropriately activated macrophages have distinctly increased cytolytic capacities, especially in the early periods of interaction, the present work makes it clear that mononuclear phagocytes from normal, unstimulated animals do have *inherent* natural killer capacity which is increased by prolonged *in vitro* interaction (Fig.).

Earlier work (Keller, 1974, 1975) showed that macrophages can promote or suppress *proliferation* of target cells *in vitro*. The present work (Tables VII and IX) confirms that the outcome of the interaction depends to a large extent on target-cell properties, and that macrophages from the same pool and in the same ratio may have a distinct cytostatic effect on some targets (rat DMBA, P815) but promote TdR incorporation by others (mouse epidermis cells, rat fibroblasts). As these capacities can differ considerably from one sample of effector cells to another, such data usually show a much larger scatter than the values representing lytic activity. Moreover, pulse-labelling of target cells after prolonged culture may be affected by a variety of uncontrolled factors; for example, target-cell proliferation can be affected by agents released from macrophages and/or killed targets such as TdR. Therefore, post-labelling techniques were not a reliable measure of DNA synthesis, especially after prolonged interaction. The present findings, which show that when cytotoxicity is consistently expressed, TdR incorporation by and proliferation of targets may either be enhanced or suppressed, strongly suggest that the macrophage effects on proliferation and viability need not be closely related.

It is evident that the predominantly phagocytic adherent cells which display potent natural killer activity are widely distributed in the host. Although their *in vitro* cytolytic capacity is selectively nullified by silica particles, they are still insufficiently characterized, and may yet prove to be heterogeneous in origin and function. Clearly, further knowledge of these cells is the first step to understanding the mechanism and significance of natural tumour resistance. Some of the more general aspects of the present findings, and especially their implications for a delimitation from other cell types with spontaneous killer capacity, have been considered elsewhere (Keller, 1977*b*; 1978).

I thank Dr K. K. Sethi, Institut für Medizinische Mikrobiologie und Immunologie der Universität, Bonn, BRD, for providing a selected IC-21 line, Dr Maurice Landy, Schweiz. Forschungsinstitut, Davos, for helpful criticism of this manuscript, and Miss R. Keist, Miss R. Ming and Miss M. Marazzi for expert technical assistance. This work was supported by the Swiss National Science Foundation (Grant 3.234.74) and the State of Zurich.

### REFERENCES

ALEXANDER, P. & EVANS, R. (1971) Endotoxin and Double-stranded RNA Render Macrophages Cytotoxic. *Nature, New Biol.*, **232**, 76.

CEROTTINI, J.-C. & BRUNNER, K. T. (1974) Cell-mediated Cytotoxicity, Allograft Rejection, and Tumor Immunity. *Adv. Immunol.*, **18**, 67.

EVANS, R. & ALEXANDER, P. (1976) Mechanisms of Extracellular Killing of Nucleated Mammalian Cells by Macrophages. In *Immunobiology of the Macrophage*. Ed. D. S. Nelson. New York: Academic Press. p. 536.

FINK, M. A. (Ed.) (1976) *The Macrophage in Neoplasia*. New York: Academic Press.

GRANGER, G. A. & WEISER, R. S. (1964) Homograft Target Cells: Specific Destruction *in vitro* by Contact Interaction with Immune Macrophages. *Science*, **145**, 1427.

GREENBERG, A. M. & PLAYFAIR, J. H. (1974) Spontaneously Arising Cytotoxicity to the P-815.Y Mastocytoma in NZB Mice. *Clin. exp. Immunol.*, **16**, 99.

HALLER, O., KIESSLING, R., ORN, A., KÄRRE, K., NILSSON, K. & WIGZELL, H. (1977) Natural Cytotoxicity to Human Leukemia Mediated by Mouse on non T-cells. *Int. J. Cancer*, **20**, 93.

HERBERMAN, R. B. & HOLDEN, H. T. (1978) Natural cell-mediated immunity. In *Advances in Cancer Research*. Eds. G. Klein & S. Weinhouse. New York: Academic Press. In press.

HERBERMAN, R. B., NUNN, M. E., HOLDEN, H. T. & LAVRIN, D. H. (1975) Natural Cytotoxic Reactivity of Mouse Lymphoid Cells against Syngeneic and Allogeneic Tumors. II. Characterization of Effector Cells. *Int. J. Cancer*, **16**, 230.

HIBBS, J. B. (1974) Discrimination between Neoplastic and Non-neoplastic Cells *in vivo* by Activated Macrophages. *J. natn. Cancer Inst.*, **53**, 1487.

HIBBS, J. B. (1976) The Macrophage as a Tumoricidal Effector Cell: a Review of *in vivo* and *in vitro* Studies on the Mechanism of the Activated

Macrophage Nonspecific Cytotoxic Reaction. In *The Macrophage in Neoplasia*. Ed. M. A. Fink. New York: Academic Press. p. 83.

HIBBS, J. B., LAMBERT, L. H. & REMINGTON, J. S. (1972) Macrophage mediated Non-specific Cytotoxicity—Possible Role in Tumour Resistance. *Nature, New Biol.*, **235**, 48.

HOLTERMANN, O. A., KLEIN, E. & CASALE, G. P. (1973) Selective Cytotoxicity of Peritoneal Leukocytes for Neoplastic Cells. *Cell. Immunol.*, **9**, 339.

KELLER, R. (1973) Cytostatic Elimination of Syngeneic Rat Tumor Cells *in vitro* by Nonspecifically Activated Macrophages. *J. exp. Med.*, **138**, 625.

KELLER, R. (1974) Modulation of Cell Proliferation by Macrophages: A Possible Function apart from Cytotoxic Tumour Rejection. *Br. J. Cancer*, **30**, 401.

KELLER, R. (1975) Major Changes in Lymphocyte Proliferation Evoked by Activated Macrophages. *Cell. Immunol.*, **17**, 542.

KELLER, R. (1976*a*) Cytostatic and Cytocidal Effects of Activated Macrophages. In *Immunobiology of the Macrophage*. Ed. D. S. Nelson. New York: Academic Press. p. 487.

KELLER, R. (1976*b*) Susceptibility of Normal and Transformed Cell Lines to Cytostatic and Cytocidal Effects exerted by Macrophages. *J. natn. Cancer Inst.*, **56**, 369.

KELLER, R. (1976*c*) Promotion of Tumor Growth *in vivo* by Anti-macrophage Agents. *J. natn. Cancer Inst.*, **57**, 1355.

KELLER, R. (1977*a*) Abrogation of Antitumor Effects of *Corynebacterium parvum* and BCG by Antimacrophage Agents. *J. natn. Cancer Inst.* **59**, 1751.

KELLER, R. (1977*b*) Mononuclear Phagocytes and Antitumour Resistance: a Discussion. In *The Macrophage and Cancer*. Eds. K. James, W. H. McBride & A. Stewart. Edinburgh Univ. Medical School. p. 31.

KELLER, R. (1978) Macrophage-mediated Natural Cytotoxicity Against Various Target Cells *in vitro*. II. Macrophages from Rats of Different Ages. *Br. J. Cancer*, **37**, 742.

KELLER, R. & JONES, V. E. (1971) Role of Activated Macrophages and Antibody in Inhibition and Enhancement of Tumour Growth in Rats. *Lancet*, ii, 847.

KIESSLING, R. & HALLER, O. (1978) Natural Killer Cells in the Mouse; an Alternative Immune Mechanism? In *Contemp. Topics in Immunology*. Ed. N. L. Warner. (In press).

KIESSLING, R., KLEIN, E., PROSS, H. & WIGZELL, H. (1975) "Natural" Killer Cells in the Mouse. II. Cytotoxic Cells with Specificity for Mouse Moloney Leukemia Cells. Characteristics of the Killer Cell. *Eur. J. Immun.*, **5**, 117.

KRAMER, J. J. & GRANGER, G. A. (1972) The *in vitro* Induction and Release of a Cell Toxin by Immune C57BL/6 Mouse Peritoneal Macrophages. *Cell. Immun.*, **3**, 88.

MAUEL, J. & DEFENDI, V. (1971) Infection and Transformation of Mouse Peritoneal Macrophages by Simian Virus 40. *J. exp. Med.*, **134**, 335.

MCLAUGHLIN, J. F., RUDDLE, N. H. & WAKSMAN, B. H. (1972) Relationship between Activation of Peritoneal Cells and their Cytopathogenicity. *J. Reticuloend. Soc.*, **12**, 293.

NATHAN, C. F., ASOFSKY, R. & TERRY, W. D. (1977) Characterization of the Nonphagocytic Adherent Cell from the Peritoneal Cavity of Normal and BCG-treated Mice. *J. Immunol.*, **118**, 1612.

NATHAN, C. F., HILL, V. M. & TERRY, W. D. (1976) Isolation of a Subpopulation of Adherent Peritoneal Cells with Anti-tumor Activity. *Nature*, **260**, 146.

NATHAN, C. F. & TERRY, W. D. (1975) Differential Stimulation of Murine Lymphoma Growth *in vitro* by Normal and BCG-activated Macrophages. *J. exp. Med.*, **142**, 887.

NELSON, D. S. (Ed.) (1976) *Immunobiology of the Macrophage*. New York: Academic Press.

NUNN, M. E., HERBERMAN, R. B. & HOLDEN, H. T. (1978) Natural Cell-mediated Cytotoxicity in Mice against Non-lymphoid Tumor Cells and Some Normal Cells. *Int. J. Cancer*, **20**, 381.

PERLMAN, P. & HOLM, G. (1969) Cytotoxic Effects of Lymphoid Cells *in vitro*. *Adv. Immun.*, **11**, 117.

SHELLAM, G. R. (1977) Gross-virus-induced Lymphoma in the Rat. V. Natural Cytotoxic Cells are Non-T Cells. *Int. J. Cancer*, **19**, 225.

SHELLAM, G. R. & HOGG, N. (1977) Gross-virus-induced Lymphoma in the Rat. IV. Cytotoxic Cells in Normal Rats. *Int. J. Cancer*, **19**, 212.

TAKASUGI, M., MICKEY, M. R. & TERASAKI, P. I. (1973) Reactivity of Lymphocytes from Normal Persons on Cultured Tumour Cells. *Cancer Res.*, **33**, 2898.

ZARLING, J. M., NOWINSKI, R. C. & BACH, F. H. (1975) Lysis of Leukemia Cells by Spleen Cells of Normal Mice. *Proc. natn. Acad. Sci. U.S.A.*, **72**, 2780.